# National analysis of the dietary index for gut microbiota and kidney stones: evidence from NHANES (2007–2018)

**DOI:** 10.3389/fnut.2025.1540688

**Published:** 2025-03-13

**Authors:** Xinzhou Yan, Xianhua Shao, Tengyue Zeng, Qijie Zhang, Junpeng Deng, Jianjun Xie

**Affiliations:** ^1^Department of Urology, Affiliated Suzhou Hospital of Nanjing Medical University, Suzhou, Jiangsu, China; ^2^Gusu School Nanjing Medical University, Suzhou, Jiangsu, China

**Keywords:** DI-GM, NHANES, kidney stones, gut microbiota, diet

## Abstract

**Background:**

Previous studies have highlighted the effects of diet and gut microbiota on the incidence of kidney stones, and the dietary index for gut microbiota (DI-GM) is a new dietary index that accurately represents the variety of gut microbiota. The current study intends to examine the potential correlation between DI-GM and kidney stones.

**Methods:**

Data from the 2007–2018 National Health and Nutrition Examination Survey (NHANES) were employed in this cross-sectional study. The history of kidney stones was assessed using a kidney conditions questionnaire. In order to examine the correlation between DI-GM and kidney stones, multivariate logistic regression was implemented. Additionally, smoothed curve fitting, subgroup analyses, and sensitivity analyses were conducted.

**Results:**

The investigation encompassed a total of 21,587 participants. After adjusting for all potential covariates, we found that DI-GM was negatively related to the incidence of kidney stones (OR = 0.96, 95% CI = 0.93–0.98, *p* = 0.0021). Compared to those in the lowest quartile, participants in the highest quartile had a lower prevalence of kidney stones (OR = 0.86, 95% CI = 0.75–0.98, *p* = 0.0252). Additionally, smoothed curve fitting revealed that DI-GM was linearly associated with the incidence of kidney stones. The results of the sensitivity analyses proved the robustness of the main analyses.

**Conclusion:**

A negative correlation between the incidence of kidney stones and DI-GM is supported by the evidence presented in this study. This finding emphasizes the potential benefits of adjusting dietary structure according to DI-GM in reducing the incidence of kidney stones. Further research should validate this discovery by employing longitudinal studies.

## 1 Introduction

As a common disease of urology, kidney stones originate from the precipitation of crystals caused by mineral oversaturation in the urine (1). Kidney stones have shown a global rise in prevalence over the past few decades, and this trend is significant across all ages, genders, and races (2). Obesity, diabetes, global warming, depression, and the overintake of salt, animal protein, sucrose, and sugar-sweetened beverages are risk factors for increased incidence of kidney stones (3, 4). Currently, surgery is the most effective treatment for kidney stones. However, the high cost of surgery, the high recurrence rate after surgery, and the severe physical and psychological burden of the patients remain challenging to resolve (5–7). Consequently, it is crucial to concentrate on the risk factors for kidney stones to establish effective strategies to mitigate the occurrence and recurrence of kidney stones.

Changes in the gut microbiota of patients with kidney stones have been identified in previous studies (8, 9). Gut microbiota dysbiosis is closely related to the environment, diet, drug use, and disease phenotypes (10, 11). A case–control study concluded that the massive use of antibiotics may lead to an increased incidence of kidney stones by causing changes in the microbiome (12). Additionally, the nutritional imbalances due to poor diet can affect the formation of kidney stones by affecting the balance of gut microbiota (13). Most recently, a novel dietary index for gut microbiota (DI-GM) was created to evaluate the correlation between gut microbiota and adult diet. By reviewing 106 articles that investigate the correlation between adult diet and gut microbiota, 14 dietary components, including 10 beneficial components and 4 unfavorable components, were selected as DI-GM components (14). Given that DI-GM was discovered to be positively correlated with indirect biomarkers of gut microbiota diversity, it is likely to become a useful instrument to reflect the impact of diet on gut microbiota. Nevertheless, the association between kidney stones and DI-GM remains unclear.

Consequently, this research employed data from the National Health and Nutrition Examination Survey (NHANES 2007–2018) to explore the correlation between DI-GM and kidney stones. After a comprehensive and rigorous analysis, our study aims to assist patients with kidney stones in maintaining a proper diet, thereby forestalling the development and recurrence of kidney stones.

## 2 Materials and methods

### 2.1 Study population

**Figure 1:**
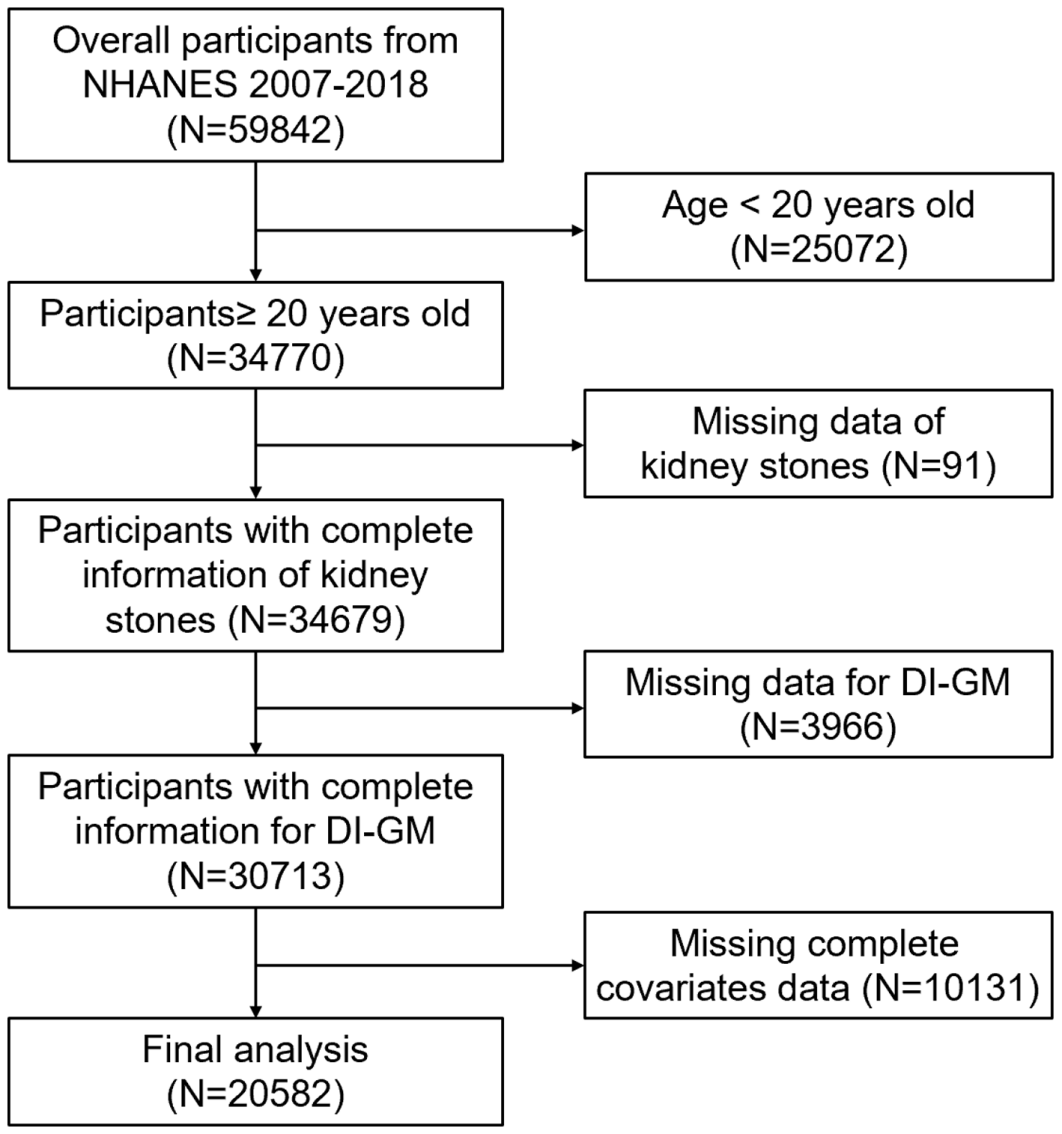
Flowchart of participant selection.

The NHANES dataset is a well-established program that is updated every 2 years to assess the nutritional and health status of participants in the United States. Each participant has offered their informed consent. Our investigation encompassed 59,842 participants from 2007 to 2018. This study’s exclusion criteria included the following: (1) age less than 20 years old (*n* = 25,072); (2) missing data of kidney stones (*n* = 91); (3) missing data about the components of DI-GM (*n* = 3,966); (4) missing data of covariates (*n* = 10,131), leaving a total of 20,582 participants for further analysis. The details of the inclusion and exclusion processes for this study were illustrated in Figure 1.

### 2.2 Exposure and outcome definitions

The outcome indicator of this study was whether the participant had kidney stones. Participants who answered affirmatively to the inquiry “Have you/has the sample person (SP) ever had kidney stones?” were detected as having a history of diagnosed kidney stones.

Following the scoring criteria created by Kase et al. (14), DI-GM was determined to consist of 14 food constituents or nutrients. Among them, 10 components, including avocados, broccoli, chickpeas, coffee, cranberries, fermented dairy, fiber, green tea, soybeans, and whole grains, were considered beneficial to gut microbiota. Score 1 for each component if consumption is equal to or above the gender-specific median; otherwise, score 0. On the contrary, 4 components, including processed meat, red meat, refined grains, and a high-fat diet (≥ 40% energy from fat), were considered unfavorable to gut microbiota. Score 0 for each component if consumption is equal to or above the gender-specific median or 40% (for a high-fat diet); otherwise, score 1. Each component’s fraction was added to determine the score of DI-GM, which ranged from 0 to 14. In general, a higher DI-GM represents enhanced gut microbiota health.

### 2.3 Covariates

In order to guarantee the robustness of the correlation between DI-GM and kidney stones, the subsequent covariates were adjusted: demographic data (age, gender, race, educational level, marital status, and poverty income ratio [PIR]), lifestyle factors (body mass index [BMI], drinking status, smoking status, and moderate recreational activity), and chronic disease conditions (diabetes and cardiovascular disease [CVD]). Age was documented as continuous values. Gender was dichotomized into male and female. Races included Mexican American, other Hispanic, Non-Hispanic White, Non-Hispanic Black, and other races. Education level was divided into below high school, high school, and above high school. Marital status was divided into never married, married/living with a partner, and widowed/divorced/separated. PIR was divided into 3 groups (<1.3, 1.3–3.5, and ≥ 3.5). BMI was divided into 3 groups (< 25, 25–30, and ≥ 30). Smoking status was defined as never, now, and former. Drinking status was determined as never, now, and former.

### 2.4 Statistical analysis

We integrated and statistically analyzed using R (version 4.2), SPSS (version 26.0), and Empowerstats (version 4.2). The absence of values for covariate variables was represented by dummy variables. The continuous variables were represented as weighted means (standard errors), while the categorical variables were expressed as unweighted counts (weighted percentages). To compare the differences between groups for continuous variables, the *F*-test was implemented, while the chi-squared test was implemented for categorical variables.

Additionally, multivariate logistic regression models were implemented to assess the correlation between DI-GM and kidney stones. Participants were grouped into Q1 (0–3), Q2 (4), Q3 (5), and Q4 (6–14) based on DI-GM scores. Specifically, model 1 was an unmodified, rudimentary model. Model 2 was adjusted by age, gender, and race. Model 3 was adjusted by age, gender, race, educational level, marital status, PIR, BMI, smoking status, drinking status, moderate recreational activity, diabetes, and CVD. Smoothed curve fitting was performed to explore the linear associations between kidney stones and DI-GM (15). Additionally, we conducted subgroup analyses to investigate the impact of a variety of covariates on the association. When a two-sided *p*-value was less than 0.05, it was deemed statistically significant.

### 2.5 Sensitivity analyses

Considering that water intake was regarded as a substantial factor in the prevalence of kidney stones (16), we further adjusted for the water intake based on model 3. In addition, we performed subgroup analyses according to gender, PIR, drinking status, BMI, and moderate recreational activity.

## 3 Results

### 3.1 Baseline characteristics

Table 1 describes the characteristics of participants in the 2007–2018 NHANES. Ultimately, 20,582 participants were enrolled in this research, consisting of 1966 participants with kidney stones and 18,616 controls, based on the inclusion and exclusion criteria. Differential characteristics were observed between participants with and without kidney stones in terms of age, gender, race, marital status, PIR, BMI, smoking status, moderate recreational activity, DI-GM, and the history of diabetes and CVD.

**Table 1:** Baseline characteristics of 20,582 participants from 2007 to 2018 NHANES.

Characteristics	Overall	Without kidney stones	Kidney stones	*p*-value
Number of participants	20,582	18,616	1966	
Age, year	47.12 (0.28)	46.50 (0.28)	52.82 (0.41)	**<0.001**
Gender				**<0.001**
Male	10,190 (49.00)	9,082 (48.29)	1,108 (55.57)	
Female	10,392 (51.00)	9,534 (51.71)	858 (44.43)	
Race				**<0.001**
Mexican American	3,095 (8.05)	2,839 (8.30)	256 (5.79)	
Other Hispanic	2089 (5.21)	1869 (5.26)	220 (4.74)	
Non-Hispanic White	9,331 (69.93)	8,202 (68.90)	1,129 (79.35)	
Non-Hispanic Black	4,077 (10.13)	3,838 (10.67)	239 (5.22)	
Other Race	1990 (6.68)	1868 (6.87)	122 (4.90)	
Education levels				0.756
< High school	4,833 (15.37)	4,345 (15.32)	488 (15.90)	
High school	4,687 (21.98)	4,251 (21.94)	436 (22.30)	
> High school	11,062 (62.65)	10,020 (62.74)	1,042 (61.80)	
Marital status				**<0.001**
Never married	3,781 (18.06)	3,608 (19.07)	173 (8.83)	
Married or living with a partner	12,310 (63.97)	11,043 (63.26)	1,267 (70.50)	
Widowed, divorced, or separated	4,491 (17.97)	3,965 (17.67)	526 (20.66)	
PIR				**0.027**
< 1.3	6,587 (21.33)	5,963 (21.52)	624 (19.60)	
≥ 1.3, <3.5	7,648 (35.28)	6,890 (34.94)	758 (38.38)	
≥ 3.5	6,347 (43.40)	5,763 (43.55)	584 (42.01)	
BMI				**<0.001**
< 25	5,906 (29.75)	5,519 (30.81)	387 (20.08)	
≥ 25, <30	6,837 (33.43)	6,177 (33.58)	660 (32.06)	
≥ 30	7,839 (36.82)	6,920 (35.61)	919 (47.86)	
Smoking status				**<0.001**
Never	11,293 (55.21)	10,352 (55.92)	941 (48.63)	
Now	4,268 (19.88)	3,871 (19.84)	397 (20.25)	
Former	5,021 (24.91)	4,393 (24.23)	628 (31.11)	
Drinking status				0.392
Never	2,859 (10.71)	2,597 (10.76)	262 (10.28)	
Now	14,963 (78.09)	13,545 (78.17)	1,418 (77.36)	
Former	2,760 (11.20)	2,474 (11.07)	286 (12.36)	
Moderate recreational activity				**0.005**
No	12,088 (53.12)	10,837 (52.66)	1,251 (57.41)	
Yes	8,494 (46.88)	7,779 (47.34)	715 (42.59)	
Diabetes				**<0.001**
No	17,463 (88.57)	16,014 (89.70)	1,449 (78.23)	
Yes	3,119 (11.43)	2,602 (10.30)	517 (21.77)	
CVD				**<0.001**
No	18,895 (93.46)	17,256 (94.18)	1,639 (86.82)	
Yes	1,687 (6.54)	1,360 (5.82)	327 (13.18)	
DI-GM	5.08 (0.03)	5.09 (0.03)	4.99 (0.05)	**0.036**
DI-GM groups				0.500
0–3	4,009 (17.60)	3,602 (17.64)	407 (17.29)	
4	4,446 (20.35)	4,026 (20.21)	420 (21.63)	
5	4,749 (22.90)	4,288 (22.83)	461 (23.56)	
≥ 6	7,378 (39.14)	6,700 (39.32)	678 (37.52)	

DI-GM, dietary index for gut microbiota; BMI, body mass index; CVD, cardiovascular disease. The DI-GM ranges from 0 to 14 and is grouped according to 0–3, 4, 5, and ≥ 6. A healthier gut microbiota is suggested by a higher DI-GM score. *p*-value less than 0.05 were bolded to indicate statistical significance.

### 3.2 Association of DI-GM with kidney stones

Table 2 showed that in model 1, the incidence of kidney stones decreased by 3% for each point increase in DI-GM (OR = 0.97, 95% CI = 0.94–0.99). The fully adjusted model maintained the significance of the aforementioned associations (OR = 0.96, 95% CI = 0.93–0.98). After grouping DI-GM, in the fully adjusted model, the incidence of kidney stones in Q4 (OR = 0.86, 95% CI = 0.75–0.98) was significantly reduced by 14% compared with Q1. Furthermore, the trend analysis indicates a correlation between DI-GM and kidney stones (*p* for trend = 0.0397).

**Table 2:** Association between DI-GM and kidney stones.

Characteristics	OR (95% CI), *p-*value		
	Model 1	Model 2	Model 3
DI-GM (continuous)	0.97 (0.94, 0.99) 0.0113	0.94 (0.91, 0.97) <0.0001	0.96 (0.93, 0.98) 0.0021
DI-GM (quartile)			
Quartile 1	Reference	Reference	Reference
Quartile 2	0.92 (0.80, 1.07) 0.2756	0.90 (0.78, 1.05) 0.1738	0.91 (0.79, 1.06) 0.2274
Quartile 3	0.95 (0.83, 1.09) 0.4876	0.92 (0.79, 1.06) 0.2282	0.94 (0.81, 1.08) 0.3914
Quartile 4	0.90 (0.79, 1.02) 0.0948	0.81 (0.71, 0.92) 0.0013	0.86 (0.75, 0.98) 0.0252
*p* for trend	0.1433	0.0019	0.0397

Model 1: no covariates were adjusted. Model 2: age, gender, and race were adjusted. Model 3: age, gender, race, educational level, marital status, PIR, BMI, smoking status, drinking status, moderate recreational activity, diabetes, and CVD were adjusted. OR, odds ratio; 95% CI, 95% confidence interval; DI-GM, dietary index for gut microbiota.

**Figure 2:**
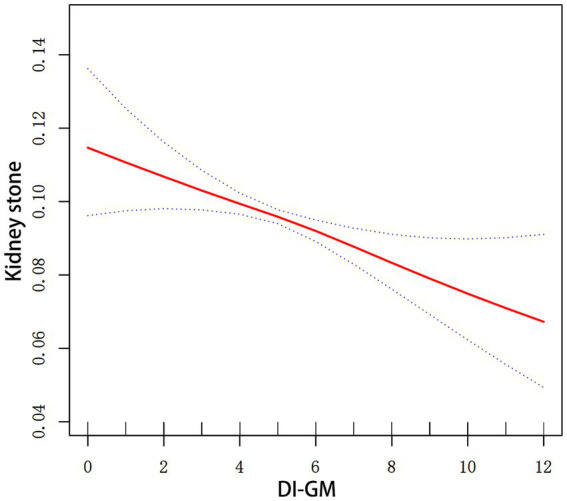
Smoothed curve fitting for DI-GM and kidney stones. Smooth curve fitting between variables is illustrated by the red line. Bands of blue color indicate the 95% CI. All covariates in Figure 2 were adjusted.

Figure 2 illustrates the association between the incidence of kidney stones and DI-GM. After adjusting for all covariates, the smoothed curve fitting results showed a linear negative association between DI-GM and the incidence of kidney stones (*P* for log-likelihood ratio test = 0.262).

### 3.3 Subgroup analysis

**Figure 3:**
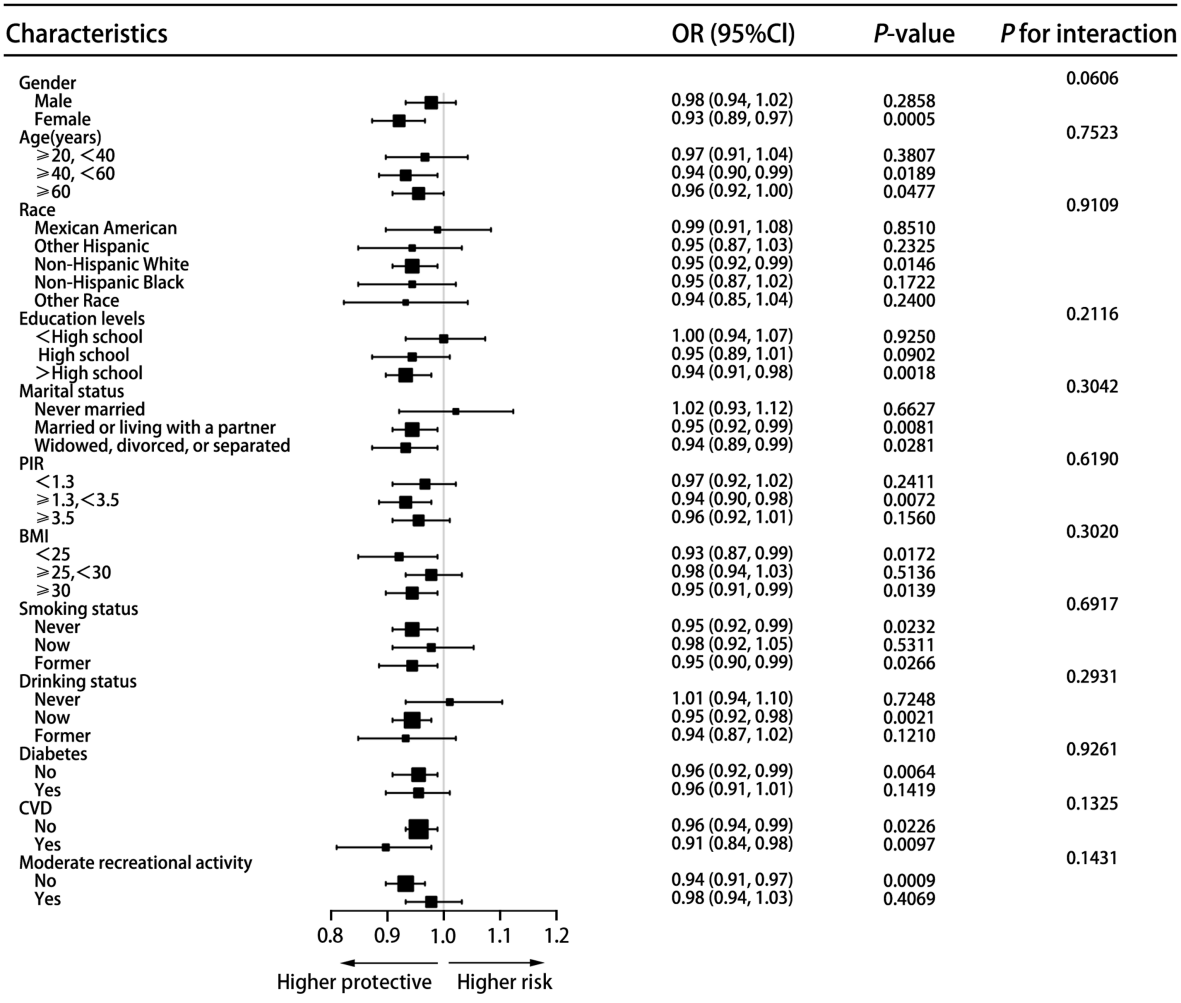
Subgroup analysis of the association between DI-GM and kidney stones.

Subgroup analysis was implemented across a variety of characteristics (Figure 3). We did not identify any substantial effect modification detected in gender, age, race, educational levels, marital status, PIR, BMI, smoking status, drinking status, diabetes, CVD, and moderate recreational activity (*p* > 0.05), despite more significant effects were observed in female participants (OR = 0.93, 95% CI = 0.89–0.97), participants aged 40–59 years (OR = 0.94, 95% CI = 0.90–0.99), participants aged ≥60 years (OR = 0.96, 95% CI = 0.92–1.00), Non-Hispanic White participants (OR = 0.95, 95% CI = 0.92–0.99), participants with a diploma above high school (OR = 0.94, 95% CI = 0.91–0.98), participants married or living with a partner (OR = 0.95, 95% CI = 0.92–0.99), participants widowed, discovered, or separated (OR = 0.94, 95% CI = 0.89–0.99), participants with a PIR ≥ 1.3 and < 3.5 (OR = 0.94, 95% CI = 0.90–0.98), participants with a BMI < 25 (OR = 0.93, 95% CI = 0.87–0.99), participants with a BMI ≥ 30 (OR = 0.95, 95% CI = 0.91–0.99), non-smokers (OR = 0.95, 95% CI = 0.92–0.99), former smokers (OR = 0.95, 95% CI = 0.90–0.99), current drinkers (OR = 0.95, 95% CI = 0.92–0.98), participants without diabetes (OR = 0.96, 95% CI = 0.92–0.99), participants without CVD (OR = 0.96, 95% CI = 0.94–0.99), participants with CVD (OR = 0.91, 95% CI = 0.84–0.98), participants lack of moderate recreational activity (OR = 0.94, 95% CI = 0.91–0.97).

### 3.4 Sensitivity analyses

The robustness of our findings was evaluated through the implementation of numerous sensitivity analyses. The association between DI-GM and kidney stones remained consistent when considering the water intake, which was highly associated with the prevalence of kidney stones, and was stable among female participants, participants with a PIR ≥ 1.3 and < 3.5, current drinkers, participants with a BMI ≥ 30, and participants without moderate recreational activity. The details of the sensitivity analyses are shown in Supplementary Tables S1–S6.

## 4 Discussion

Our study verified that the incidence of kidney stones was inversely correlated with increases in DI-GM. The study population was divided into four groups based on the quartiles of DI-GM scores. The fully adjusted model demonstrated a substantial decrease in the incidence of kidney stones in Q4, as compared to Q1. The smoothed curve fitting results showed that DI-GM was linearly associated with the incidence of kidney stones. In addition, the robustness of these findings was further confirmed by sensitivity analyses.

Oxalate is the most common component of kidney stones, and the effects of gut microbiota in degrading oxalate have historically been emphasized (17). Initially, an intestinal bacterium named *O. formigenes* was found to be responsible for the degradation of oxalate (18). Subsequently, 18 other gut microbes were identified as capable of degrading oxalate, indicating the possibility of preventing kidney stones by introducing specific bacteria into the human intestine (19). In addition to degrading oxalate, the altered gut microbiota can influence kidney stone formation by promoting lipid metabolism or leading to compromised integrity of the intestinal barrier, thereby enhancing paracellular absorption of oxalate and inducing renal inflammation (20, 21). To verify the effect of gut microbiota on kidney stone formation, a study transplanted feces from patients with kidney stones into rats and found that overgrowth of *Bacteroidota* had a strong influence on the formation of calcium oxalate kidney stones by influencing the intestinal barrier function, hyperoxaluria, and renal inflammation (22). In another study, rats with high dietary oxalate exhibited gut microbiota disturbances, while transplanting microbes from healthy rats effectively reduced CaOx crystal depositions by promoting the expression of intestinal barrier proteins and oxalate transporters (23). Additionally, a two-sample Mendelian randomization study confirmed the causal relationship between kidney stones and gut microbiota (24). In general, these studies emphasized the association between gut microbiota diversity and kidney stone formation, providing the possibility of preventing kidney stone formation by maintaining the stability of gut microbiota.

Diet can substantially influence the gut microbiota (25, 26) and then influence the development and recurrence of kidney stones. The intakes of salt, animal proteins, oxalate, calcium, fruit, vegetables, legumes, and water were found to be highly associated with gut microbiota diversity and the incidence of kidney stones (13). However, there is a lack of studies supporting the strong correlation between dietary intake and kidney stone-associated dysbiosis of the gut microbiota. Dietary index is a tool used to evaluate the nutritional health of an individual’s diet based on a variety of dietary components. Compared to a single dietary component, a dietary index can provide a multidimensional and comprehensive assessment of dietary quality that helps people better identify potential nutritional problems and guides them to take appropriate steps to improve their diets (27). The newly created dietary index DI-GM can serve as an indicator of the correlation between gut microbiota and diet. The changes of DI-GM suggest changes in dietary structure, which in turn influence the diversity of gut microbiota and are closely related to various pathophysiological processes. In a recent cross-sectional study, the researchers discovered a negative correlation between DI-GM and depression, together with the mediating function of phenotypic age and BMI (28). Another study confirmed the negative correlation between DI-GM and the risk of accelerated aging, with BMI mediating this association (29). Our study identified a negative correlation between DI-GM and kidney stones, but the specific biological mechanisms involved remains unclear.

The potential influence of certain components of DI-GM on the gut microbiota and their regulatory effect on kidney stone formation have been noticed for a long time. Moderate coffee intake was found to be associated with higher gut microbiota diversity and richer beneficial flora (30). Several population-based studies have found the beneficial effect of coffee intake on preventing kidney stones (31, 32). Mechanistically, coffee intake can reduce the release of antidiuretic hormone and thus exert a diuretic effect. Furthermore, caffeine was found to inhibit kidney stone formation by promoting the translocation of annexin A1 to reduce the adhesion of calcium oxalate crystals to renal tubular epithelial cells (33). Cranberry is another component that is beneficial to the gut microbiota. It has been found to modulate the composition of the gut microbiota and increase the content of Bifidobacterium, which can inhibit kidney stones by degrading oxalate (34, 35). Additionally, the green tea polyphenol was found to exert protective effects on chronic diseases associated with oxidative stress by promoting the growth of beneficial flora (36). An *in vivo* experiment revealed that tea polyphenol intake suppressed the formation of kidney stones by improving oxidative stress (37). However, the specific mechanism of how these components inhibit kidney stone formation by maintaining gut microbiota stability remains unclear.

As far as we are aware, this study is the first to confirm a negative relationship between gut microbiota-related DI-GM and kidney stones. Changes in the DI-GM suggest changes in dietary patterns, which in turn influence the diversity of the gut microbiota and are linked to the prevalence of kidney stones. The gut-kidney axis is typically believed to be influenced by reduced gut microbiota diversity, which may result in kidney stones (38). This understanding may offer potential implications for preventing kidney stones. However, this correlation requires additional investigation through the implementation of more rigorously designed clinical and fundamental research studies that employ substantial samples.

Nevertheless, the current investigation contains numerous constraints. Firstly, considering that this study was conducted using cross-sectional data, the establishment of a causal relationship between kidney stones and DI-GM is impossible, and the results may be susceptible to selection bias. Further prospective cohort studies and Mendelian randomization studies are needed to establish causality. Secondly, it is possible that the correlation between DI-GM and kidney stones in other countries may differ from the American population used in this study due to the influence of cultural and regional differences on dietary composition. Furthermore, it is uncertain whether the negative correlation between DI-GM and kidney stones remains consistent across different types of kidney stones, since diet affects different types of kidney stones to varying degrees. Thirdly, the DI-GM assessment was conducted using self-reported 24-h dietary records, which may have increased recall bias. Additionally, the non-specific indicator of gut microbiota diversity, urinary enterolignans, failed to fully capture the complexity of the gut microbiota. Lastly, the prevalence of kidney stones and the gut microbiota diversity can also be influenced by certain underlying diseases and drug use, especially antibiotics. Nevertheless, the consequences of these factors have not been entirely eradicated in this study.

## 5 Conclusion

In conclusion, a substantial negative correlation between the prevalence of kidney stones and the newly proposed DI-GM is suggested by the current study. Dietary interventions designed in accordance with the DI-GM score may help reduce the incidence of kidney stones, given the robust correlation between diet, gut microbiota, and kidney stones.

## Data Availability

The original contributions presented in the study are included in the article/supplementary material, further inquiries can be directed to the corresponding authors.
